# Does Grandparenting Worsen Loneliness? The Conditional Role of Care and Activity

**DOI:** 10.1093/geroni/igaf122.4103

**Published:** 2025-12-31

**Authors:** In Jeong Hwang, Joseph Svec

**Affiliations:** Iowa State University, Ames, Iowa, United States; St. Joseph’s University, Patchogue, New York, United States

## Abstract

Research on grandparenting has found seemingly contradictory patterns: grandparental childcare is often linked to poorer well-being, but grandparenting is also associated with reduced loneliness. These inconsistencies highlight that grandparenting is not a monolithic experience. However, little attention has been paid to the different profiles of grandparent–grandchild interaction, as prior work has typically treated it as either caregiving or leisure. This study identifies profiles of grandparenting—care only, activity only, and care combined with activity—and examines how these relate to grandparents’ loneliness. Using the last four waves of the Health and Retirement Study (2016, 2018, 2020, 2022; N = 7,586), we estimate random-effects panel models with clustered standard errors. We find that providing care in the absence of activities was associated with significantly greater loneliness, suggesting that purely instrumental caregiving can be burdensome. In contrast, engaging in activities with grandchildren was consistently linked to reduced loneliness, with the strongest protective effect at moderate activity levels. At higher activity levels, caregiving embedded in shared interactions buffers the negative effect of caregiving alone, and in some cases caregivers report less loneliness compared to non-caregiver grandparents. These findings suggest that the well-being consequences of grandparenting are conditional on the balance between caregiving and activity. This study underscores the importance of supporting shared, meaningful activities to strengthen intergenerational ties and reduce loneliness among older adults.

